# Characterization of 3-Dimensional Printing and Casting Materials for use in Magnetic Resonance Imaging Phantoms at 3 T

**DOI:** 10.6028/jres.125.028

**Published:** 2020-09-15

**Authors:** B. E. Yunker, K. F. Stupic, J. L. Wagner, S. Huddle, R. Shandas, R. F. Weir, S. E. Russek, K. E. Keenan

**Affiliations:** 1 National Institute of Standards and Technology, Boulder, CO 80305, USA; 2 University of Colorado-Denver/Anschutz, Aurora, CO 80045, USA

**Keywords:** 3D printing, medical imaging, MRI, phantom, polymer

## Abstract

Imaging phantoms are used to calibrate and validate the performance of magnetic resonance imaging (MRI) systems. Many new materials have been developed for additive manufacturing (three-dimensional [3D] printing) processes that may be useful in the direct printing or casting of dimensionally accurate, anatomically accurate, patient-specific, and/or biomimetic MRI phantoms. The T1, T2, and T2* spin relaxation times of polymer samples were tested to discover materials for use as tissue mimics and structures in MRI phantoms. This study included a cohort of polymer compounds that was tested in cured form. The cohort consisted of 101 standardized polymer samples fabricated from: two-part silicones and polyurethanes used in commercial casting processes; one-part optically cured polyurethanes used in 3D printing; and fused deposition thermoplastics used in 3D printing. The testing was performed at 3 T using inversion recovery, spin echo, and gradient echo sequences for T1, T2, and T2*, respectively. T1, T2, and T2* values were plotted with error bars to allow the reader to assess how well a polymer matches a tissue for a specific application. A correlation was performed between T1, T2, T2* values and material density, elongation, tensile strength, and hardness. Two silicones, SI_XP-643 and SI_P-45, may be usable mimics for reported liver values; one silicone, SI_XP-643, may be a useful mimic for muscle; one silicone, SI_XP-738, may be a useful mimic for white matter; and four silicones, SI_P-15, SI_GI-1000, SI_GI-1040, and SI_GI-1110, may be usable mimics for spinal cord. Elongation correlated to T2 (p = 0.0007), tensile strength correlated to T1 (p = 0.002), T2 (p = 0.0003), and T2* (p = 0.003). The 80 samples not providing measurable signal with T1, T2, T2* relaxation values too short to measure with the standard sequences, may be useful for MRI-invisible fixturing and medical devices at 3 T.

## 1 Introduction

Magnetic resonance imaging (MRI) has become an invaluable medical diagnostic tool in many applications [1-4]. MRI images are formed by placing a patient or material sample into a highly uniform magnetic field (B0) along the long (*z*) axis of the scanner to align the proton spins of the tissue or material parallel with the field [5]. Typical clinical scanners, using superconducting magnets, operate at field values of 1.5 T and 3 T, with 7 T clinical systems now becoming available. New low-field MRI scanners are also becoming available with fields below 100 mT. Additional magnetic field gradients (*G_x_*, *G_y_*, *G_z_*) are applied in the *x*, *y*, and *z* directions for spatial encoding, along with transient radio-frequency (RF) magnetic field pulses, of amplitude B1, to excite the spins and tip them away from their equilibrium position. The B1 pulse, for which the frequency is at or near the precessional frequency of the nuclear spin being imaging, is generated from transverse-mounted RF coils. Most often, MRI detects the induced field produced by precessing proton spins on water and fat with a resonant frequency $\omega_{0}=\gamma_{p}B_{0}$, where $\gamma_{p}$ is the gyromagnetic ratio of the proton of interest, and resonant frequencies, ${f_{0}=\omega }_{0}/2\pi$, are approximately 63.9 MHz, 127.8 MHz, and 298 MHz for 1.5 T, 3.0 T, and 7 T, respectively. The RF pulses, along with the gradient pulses, are choreographed into complex sequences to form the desired image. The RF pulses tip the proton spin moment by an angle α, from parallel to the B0 field to typically α = 90° (perpendicular to the B0 field) or α = 180° (antiparallel to B0, −*z* direction). The proton spin magnetization, $M\left(t\right)$, will precess about B0 and relax back to its equilibrium value with different exponential decay rates, which are a function of material, molecular interactions, field strength, and temperature. The image contrast for many pulse sequences is set, to a large extent, by the relaxation times, denoted T_1_, T_2_, and T_2_*, of different materials and tissues.^1^1 The MRI signal can be sensitive to several other material parameters, such as proton density, diffusion, electrical conductivity, and magnetic susceptibility, depending on the type of pulse sequence used. In this paper, we are only focusing on spin relaxation times.

T_1_ is the longitudinal relaxation time, which characterizes the time it takes to go from the initial excited *z*-axis magnetization, $M_{zi}$, to the equilibrium *z*-axis magnetization, $M_{0}$:

Mzt=Mzi+M0-Mzi1-e-tT11

T_2_ is the transverse relaxation time and characterizes the decay of the transverse magnetization$\;M_{xy}=M_{x}\hat{x}+M_{y}\hat{y}$ to zero in the absence of any extrinsic dephasing effects:

Mxyt=Mt0e-iω0te-tT22

where $M_{xy}=M_{x}+iM_{y}$ is the complex transverse magnetization, and $M_{t0}$ is the initial magnetization just after the excitation pulse. T_2_* is the total dephasing time that includes effects due to intrinsic material properties (T_2_ relaxation) plus extrinsic field inhomogeneities due to sample or scanner created field inhomogeneities. From an operational perspective, T_2_ describes the dephasing component that cannot be rephased by a spin echo sequence that incorporates rephasing pulses. T_2_* is always less than T_2_ and is a function of the nonlocal environment. Since T_1_ relaxation involves energy lost to adjacent macromolecules (spin-lattice), and T_2_ decay involves angular momentum transfer to adjacent spins (spin-spin), there may be material properties such as density, elongation, tensile strength, or hardness that correlate with T_1_, T_2_, and T_2_* values, as they are sensitive to molecular structure and interactions [5].

MRI calibration phantoms are used to assess stability in MRI scans over time, as well as establish consistency between manufacturers and models of scanners. These phantoms generally include accurately located vials of specific chemical solutions [6, 7]. The fabrication of MRI phantoms historically involves machining and casting of large plastic components using manual and automated machining equipment. This approach involves considerable labor and machine time costs and feature detail that is limited to tool size and range of motion. The use of 3D printing for general medical applications is well documented, with multiple-material capability, improving accuracy, and decreasing costs of 3D printing technology rapidly developing [8-18]. These trends open opportunities to fabricate highly detailed calibration phantoms, as well as finely detailed patient-specific anatomical models for surgical planning and training. There are several studies describing the use of 3D printing materials and technology for MRI phantom applications [19-21].

In previous research, small numbers of two-part silicone and polyurethane polymers have been imaged with computed tomography (CT), MRI, and ultrasound [22, 23]. The results suggested that some of the materials might be suitable for use in MRI/CT/ultrasound imaging phantoms and mechanical test models. Additionally, the viscosities of the uncured polymer components appeared to be compatible with 3D printing through sub-millimeter-size nozzles.

This research was performed to discover or predict materials with T_1_, T_2_, and T_2_* relaxation values similar to human tissues for use as mimics, or, materials with no measurable relaxation values for use as MRI-compatible support structures. This study did not investigate the material’s dielectric or magnetic susceptibility properties, which can also influence the MRI signal by distorting the RF and magnetic fields.

## 2 Methods

A list of the physical properties of candidate materials was compiled from manufacturer data sheets. The documented tissue values for T_1_, T_2_, and T_2_* at 3 T were obtained from peer-reviewed journal papers. The selection criteria for sample fabrication included availability within the project schedule, cost within available funding, ease and speed of fabrication, and toxicity that could be accommodated with standard protective gear and large room ventilation.

Samples of 3D printing materials were fabricated as 10 mm × 15 mm × 20 mm (± 0.01 mm) cuboids. Standard samples of one-part ultraviolet-cured polyurethanes were printed with a FormLabs (Somerville, MA) (www.formlabs.com) Form 2 stereolithographic laser (SLA) printer.^2^2 Certain commercial equipment, instruments, and/or materials are identified in this report in order to adequately specify the experimental procedure. Such identification does not imply recommendation or endorsement by the National Institute of Standards and Technology, nor does it imply that the equipment and/or materials used are necessarily the best available for the purpose. Standard samples of one-part polyurethanes and fused deposition modeling (FDM) materials were procured from third-party 3D printing fabricators Protocam (Allentown, PA) (www.protocam.com), Protogenic (Westminster, CO) (www.tenere.com), Protolabs (Maple Plain, MN) (www.protolabs.com), and Sculpteo (San Leandro, CA) (www.sculpteo.com). Since printer manufacturers offer materials optimized for each printer model and offer some compatibility with third-party materials, this sourcing strategy gave access to materials and chemistries from all major suppliers (3Dsystems, ALM, Carbon, Carbon Resin, DSM Somos, EOS, and Stratasys).

Samples of cured silicones were cut down to 20 mm × 25 mm × 5 mm (± 2 mm) from precast material obtained from Silicones, Inc. (High Point, NC) (www.silicones-inc.com), and from Smooth-On, Inc. (Macungie, PA) (www.smooth-on.com). A sample of a two-part polyurethane from Huntsman (Woodlands, TX) (www.freeman.com was also cut down. These sample sizes were chosen to ensure capture of at least one 4 mm coronal slice with enough protons for a detectable signal within a 6 mm diameter region of interest (ROI). Since T_1_ and T_2_ are intrinsic characteristics of materials and tissues, the exact dimensions of the samples were not relevant, provided enough protons were captured to emit a signal measurably above the scanner noise floor. T_2_* measurements are affected by the sample geometry, since magnetic susceptibility variations can lead to additional field inhomogeneity. Care needs to be taken when associating T_2_* values with material properties, particularly for materials with longer T_2_* times, where the spin dephasing may be dominated by system and geometry inhomogeneities.

The test samples were placed in a 31 day commercial pill organizer (www.amazon.com), which exhibited no MRI signal at 3 T with the sequences used for testing. An Agilent 7T310 (Santa Clara, CA) (www.agilent.com) preclinical scanner operating at 3 T was used for the measurement of T_1_, T_2_, and T_2_* in this study. The scanner was equipped with a 140 mm quadrature birdcage RF coil from Doty Scientific (Columbia, SC) (www.dotynmr.com) with an isocenter accommodating 12 samples within the uniform field of view. To maintain coil loading between groups, one pill well in each group of 12 samples was filled with deionized (DI) water. The scanner used for this study was kept at a high level of calibration for quantitative imaging phantom development at the National Institute of Standards and Technology (NIST).

The MRI sequences listed in Table A1 were used to obtain T_1_, T_2_, and T_2_* values using a single 4 mm coronal (*x*-*z* plane) slice. The RF coil was retuned and matched at 50 ohms for the imaging of each section to accommodate the variable loading of samples.

The T_1_, T_2_, and T_2_* relaxation times of the samples were obtained from series of magnitude images obtained from conventional inversion recovery, spin echo, and gradient echo sequences, respectively [24]. The inversion recovery sequence tips the spins by α = 180° and then waits an inversion time (TI) before tipping the spins into the transverse plane and detecting the induced signal. The spin echo sequence tips the spins by α = 90° and then, after a time TE/2, applies a refocusing pulse and records the signal at echo time TE. For the gradient echo sequence, the spins are tipped α = 90°, and then the signal is read at a time TE without any refocusing. For T_1_, the inversion time, TI, was varied, and for T_2_ and T_2_*, the echo time, TE, was varied. The relaxation times were computed using the Python-based NIST *PhantomViewer* software application (www.github.com/NIST/PhantomViewer) by fitting the observed signal, S, to the models described in Eq. (3) for T_1_ and Eq. (4) for T_2_ and T_2_*. Standard nonlinear least squares fit routines (Levenberg Marquardt) from the Scipy library (www.scipy.org/scipylib) were used.

STI=A1-Be-TIT1.3

STE=S0e-TET2 .4

The initial parameter guesses for the nonlinear least squares fitting were T_1guess_ = TI_amin_/ln2 for T_1_ and T_2guess_ = 200 ms for T_2_ fitting, where TI_amin_ is the value of TI that gives a minimum signal. The computed relaxation values and error bars were plotted alongside tissue relaxation values and error bars taken from literature.

To uncover predictive relationships, the measured T_1_, T_2_, and T_2_* values were plotted against the physical properties listed by the manufacturers, in addition to a least squares statistical analysis performed using the JMP (Cary, NC) (www.jmp.com) software application. Materials with *R*^2^ > 0.5 and *p* < 0.05 were considered statistically significant for the purposes of this exploratory study.

## 3 Results

The properties of over 1200 castable and printable materials were reviewed for use as mimics for human tissues. Many of the human tissue values are cited by the original sources in this study [25-43] and are summarized by Bojorquez *et al*. [33]. The T_1_ and, T_2_ values for tissues found in literature are provided in Table A2 sorted alphabetically.

Materials with signals below the noise level and T_1_, T_2_, T_2_* values too short to measure using standard pulse sequences, are provided in Table S1 along with their physical characteristics.

**Fig. 1:**
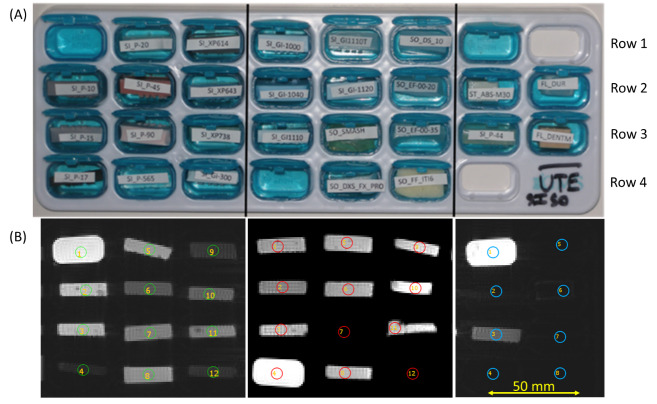
(A) Pill organizer loaded with labeled material samples in pill wells and (B) corresponding coronal gradient echo (T_2_*) images at TE 2.9 ms (sections 1-3). Sample list: Row 1 - water, SI_P-20, SI_XP614, SI_GI1000, SI_GI1110T, SO_DS_10, water, empty; Row 2 - SI_P-10, SI_P-45, SI_643, SI_GI-1040, SI_GI-1120, SO_EF-00-20, ST_ABS-M30, FL_Dur; Row 3 - SI_P-15, SI_P-90, SI_XP738, SI_GI1110, SO_SMASH, SOEF-00-35, SI_P-44, FL_DENTM; Row 4 - SI_P-17, SI_P-565, SI_GI-300, water, SO_DXS_FX_PRO, SO_FF_ITI6, empty.

The T_1_, T_2_, and T_2_* values of the samples that had measurable values are listed in Table A3 for materials, sorted by increasing T_1_ value, along with their respective physical properties. The samples exhibiting measurable signal are shown in a photo of the tray in Fig. 1.

The T_1_, T_2_, and T_2_* values of the materials and values of human tissue are plotted in Fig. 2 (a)-(c), with error bars where error data were available. Error bars were truncated on the top chart margin in favor of reducing plot detail. Since all error bars are symmetric, the truncated upper values can be deduced from the lower error bar.

**Figure:**
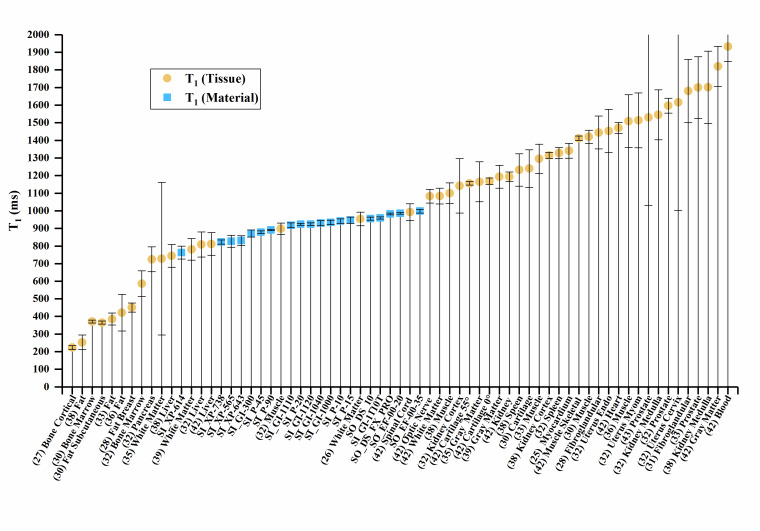
(a)

**Figure:**
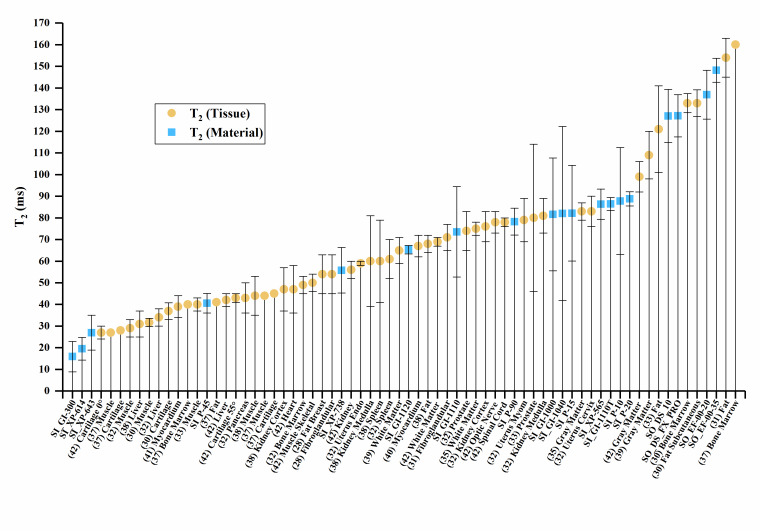
(b)

**Figure:**
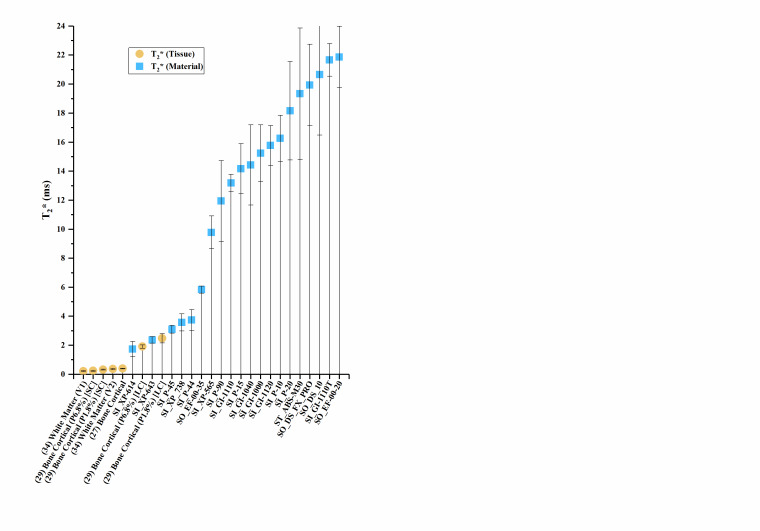
(c)

**Fig. 2:** (a) Sample material T_1_ values compared to human tissues. (b) Sample material T_2_ values compared to human tissues. (c) Sample material T_2_* values compared to human tissues, where SC indicates the short compartment of T_2_*, and LC indicates the long compartment T_2_*, Px.x% is porosity percent, and (Vx) is Volunteer #.

The T_1_ vs. T_2_, T_1_ vs. T_2_*, and T_2_ vs. T_2_* values are plotted in Fig. 3 (a)-(d) overlaid with values of human tissues, with error bars where error data were available. Error bars were truncated on the top chart margin in favor of reducing plot detail. Since all error bars are symmetric, the truncated upper values can be deduced from the lower error bar.

**Figure:**
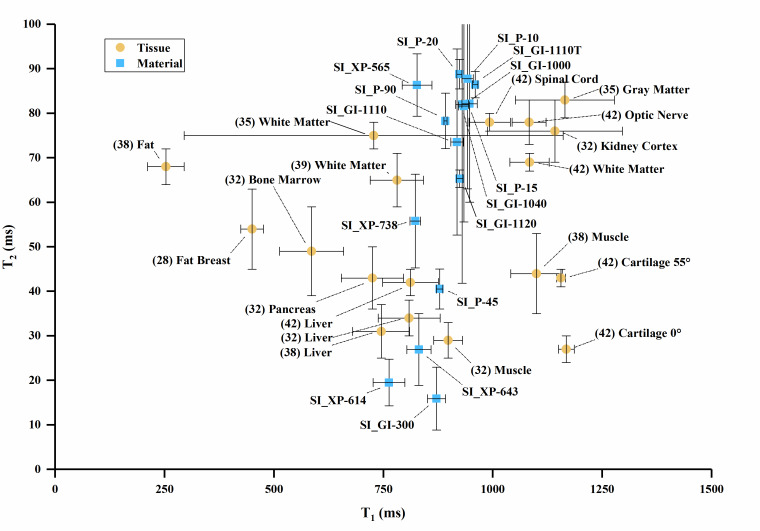
(a)

**Figure:**
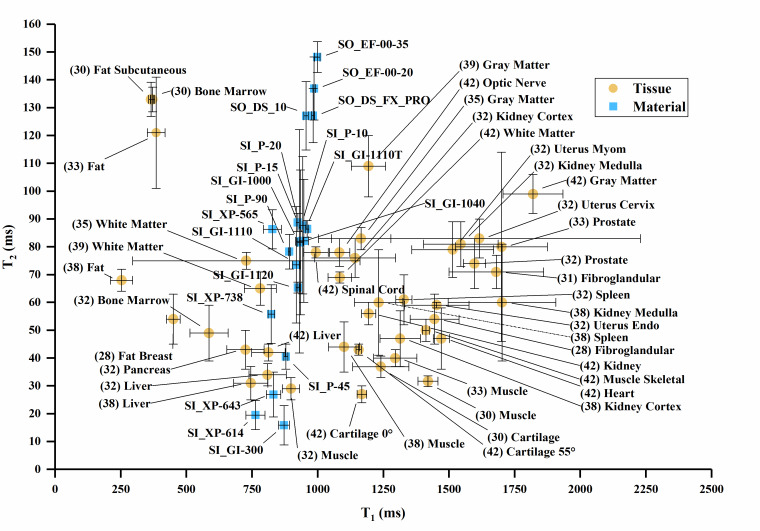
(b)

**Figure:**
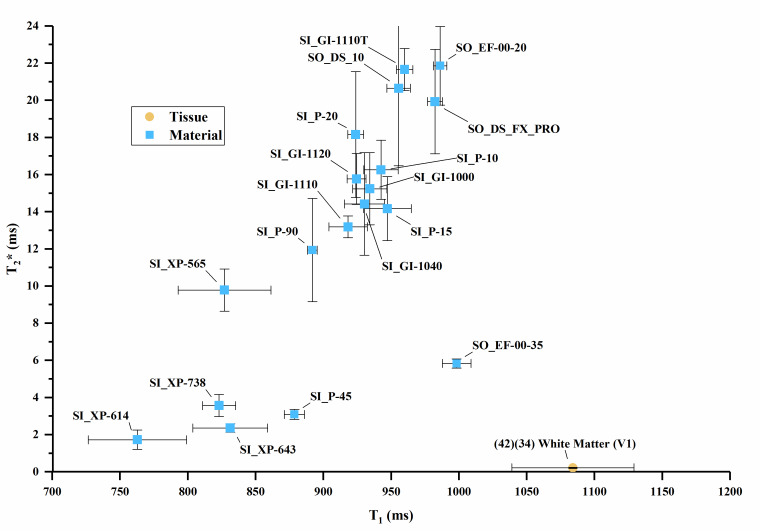
(c)

**Figure:**
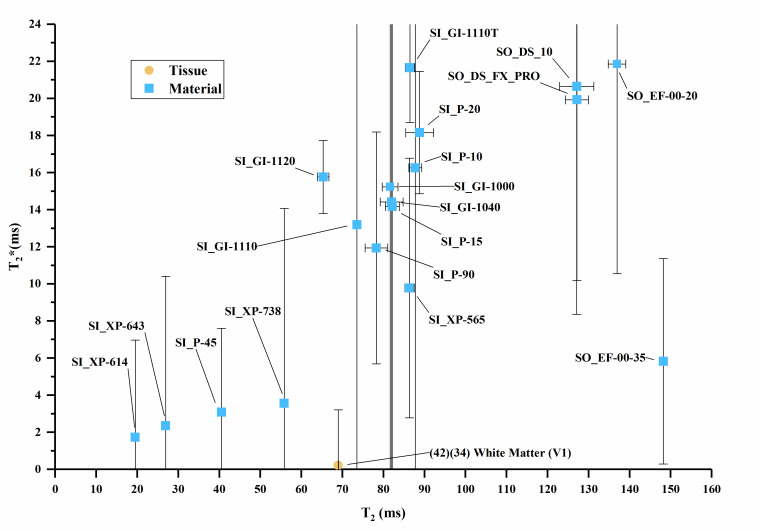
(d)

**Fig. 3:** (a) T_1_ vs. T_2_ values for sample materials near human tissue values. (b) T_1_ vs. T_2_ values for sample materials compared to many human tissues. (c) T_1_ vs. T_2_* values for sample materials. (d) T_2_ vs. T_2_* values for sample materials. Note: (1) The white matter data point combines T_2_* and T_1_ data from two authors in Table A3 [34, 42]. (2) Cortical bone values plot below the axis scale [T_1_ = 223 ms, T_2_ = 11 ms, T_2_* = 0.39 ms] [27].

A summary of the statistical analysis is provided in Table A4 and in Figs. S1-12, including *R*^2^, *p* values, and fit model. Elongation correlated to T_2_ (*p* = 0.0007), and tensile strength correlated to T_1_ (*p* = 0.002), T_2_ (*p* = 0.0003), and T_2_* (*p* = 0.003). Density and hardness did not correlate to relaxation values.

## 4 Discussion

There were several materials for which the measured T_1_ and T_2_ values were near tissue values. The materials with measurable T_1_, T_2_, and T_2_* values using the available MRI sequences were cast silicones, although T_1_, T_2_, and T_2_* were not predicted by silicone condensation or addition cure chemistry. The statistical analysis showed that T_2_ increased with increasing material elongation, suggesting that increased molecular distance reduced the opportunity for spin-spin energy transfer. The other significant correlation was that T_1_, T_2_, and T_2_* decreased with increasing tensile strength, suggesting that increasing molecular rigidity increased spin-lattice and spin-spin coupling. T_1_, T_2_, and T_2_* were not predicted by density or hardness.

The relaxation values for the 3D printed thermoplastics and polyurethanes were not measurable with the scanner and sequences used in the study due to short decay times. Future research will be conducted on a scanner capable of running ultrashort (UTE) sequences to determine if the “no signal” and “no fit” samples from this research have use as mimics for short T_2_* tissues. These low-signal materials might be used in the fabrication of very precise MRI-compatible fixtures and accessories such as RF and gradient coil housings, magnetic probe holders, patient head and body alignment fixtures, or functional MRI actuators to name a few examples.

The study was limited by several factors. Only the four material characteristics evaluated in this study were consistently reported by all manufacturers. Few peer-reviewed papers reported T_1_, T_2_, and T_2_* for the same tissue because most papers focused on one or two of the three relaxation times due to lack of instrumentation. The intimate details of each material’s chemistry and processing were not known, so the size and mobility of the molecules were not known. Future chemistry knowledge might explain some apparent groupings in the density plots and justify separate fits in future analyses.

Given this study was performed at 3 T and that tissue and material T_1_, T_2_, and T_2_* relaxation times change with field strength, it is expected that the relative material/tissue values would be different at 1.5 T and 7 T.

## 5 Conclusion

Two silicones, SI_XP-643 (T_1_ = 831.3 ms, T_2_ = 26.9 ms) and SI_P-45 (T_1_ = 878.6 ms, T_2_ = 40.5 ms), may be usable mimics for reported liver values; one silicone, SI_XP-643 (T_1_ = 831.3 ms, T_2_ = 26.9 ms), may be a useful mimic for muscle; one silicone, SI_XP-738 (T_1_ = 823.0 ms, T_2_ = 55.8 ms), may be a useful mimic for white matter; and four silicones, SI_P-15 (T_1_ = 947.2 ms, T_2_ = 82.1 ms), SI_GI-1000 (T_1_ = 934.2 ms, T_2_ = 81.2 ms), SI_GI-1040 (T_1_ = 930.26 ms, T_2_ = 81.99 ms), and SI_GI-1110 (T_1_ = 918.2 ms, T_2_ = 73.5 ms), may be usable mimics for spinal cord [32, 38, 39, 42]. Elongation correlated to T_2_ (*p* = 0.0007), and tensile strength correlated to T_1_ (*p* = 0.002), T_2_ (*p* = 0.0003), and T_2_* (*p* = 0.003). The 80 samples not providing measurable T_1_, T_2_, and T_2_* relaxation times with the standard sequences used in this study should be good candidates for MRI-compatible fixtures and medical devices at 3 T.

## Figures and Tables

**Fig. S1:**
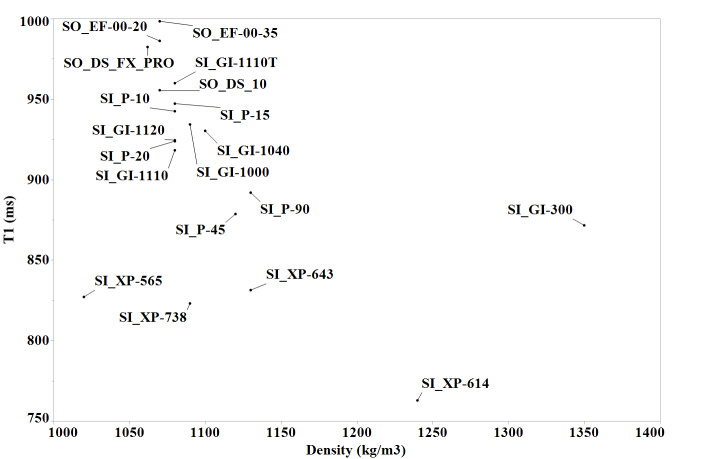
T_1_ vs Density.

**Fig. S2:**
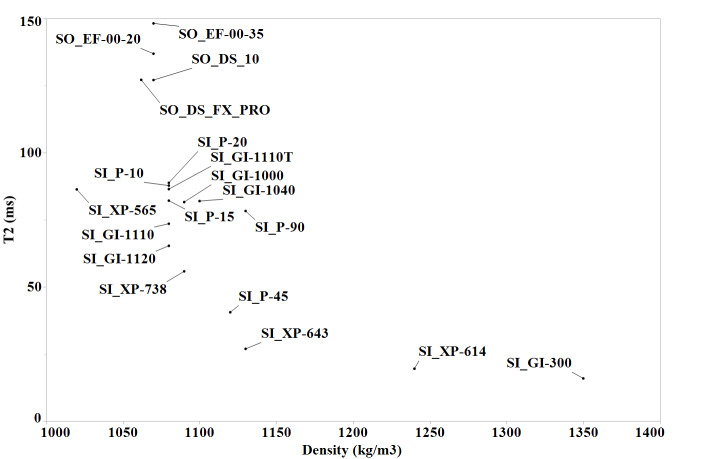
T_2_ vs Density.

**Fig. S3:**
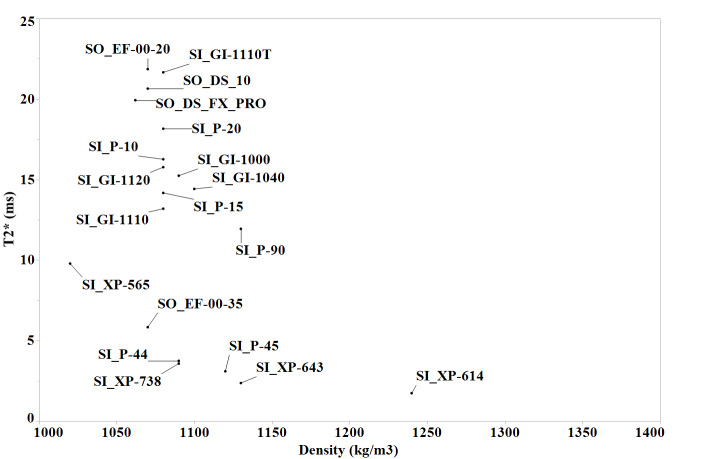
T_2_* vs Density.

**Fig. S4:**
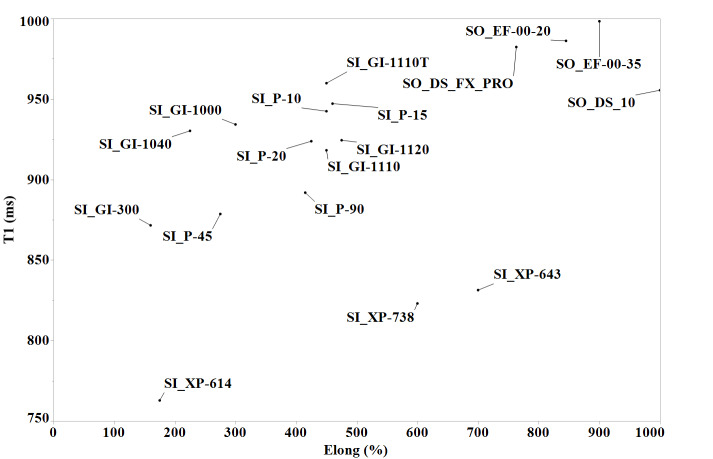
T_1_ vs Elongation.

**Fig. S5:**
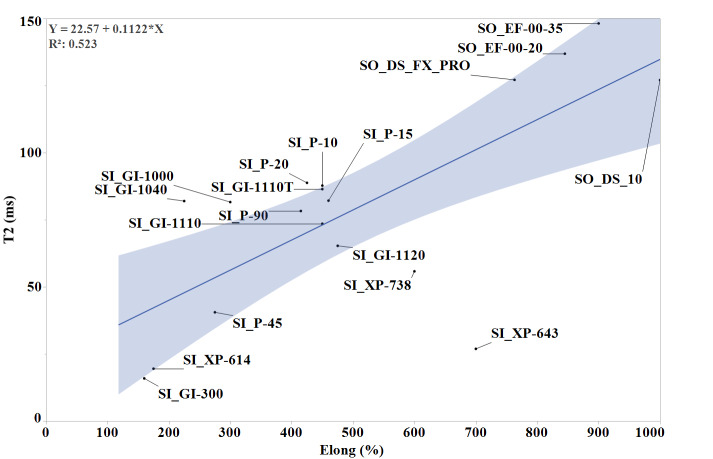
T_2_ vs Elongation - T_2_ increases with increasing Elongation (p = 0.0007).

**Fig. S6:**
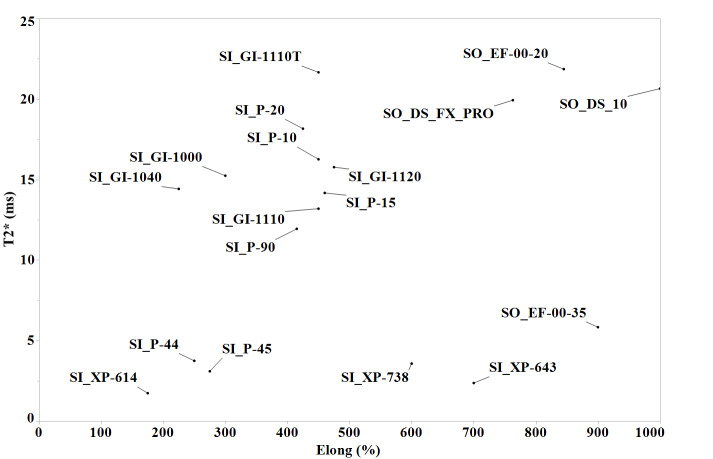
T_2_* vs Elongation.

**Fig. S7:**
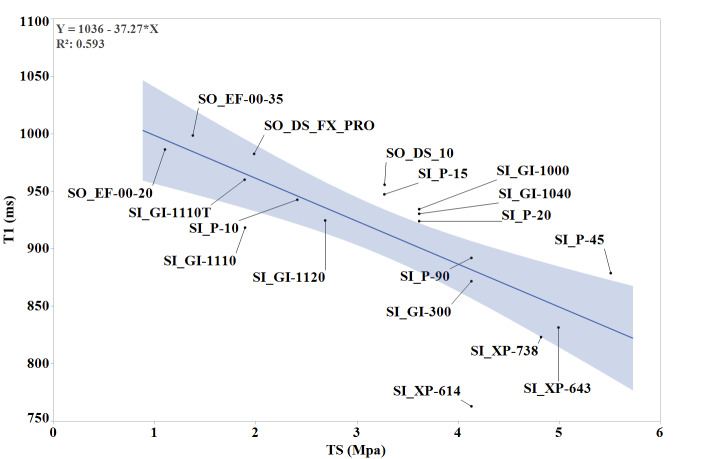
T_1_ vs Tensile Strength - T_1_ decreases with increasing Tensile Strength (p = 0.002).

**Fig. S8:**
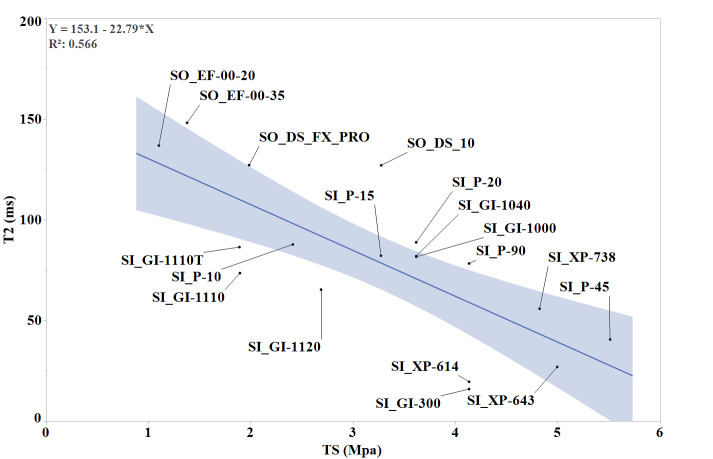
T_2_ vs Tensile Strength - T_2_ decreases with increasing Tensile Strength (p = 0.0003).

**Fig. S9:**
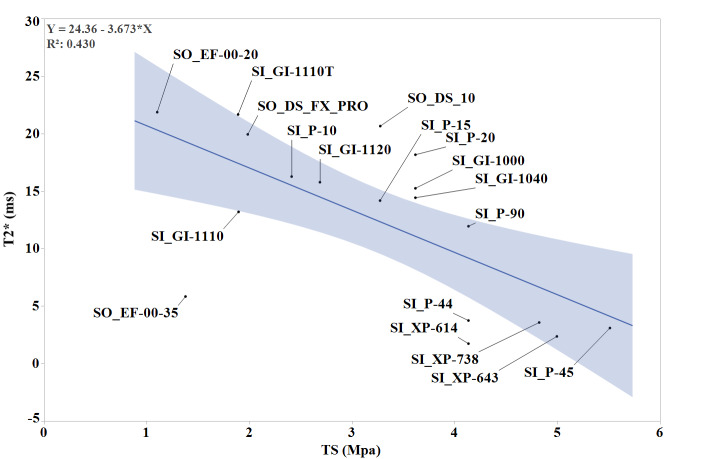
T_2_* vs Tensile Strength (p =0.0031) - T_2_* decreases with increasing Tensile Strength and nearly meets the significance criteria.

**Fig. S10:**
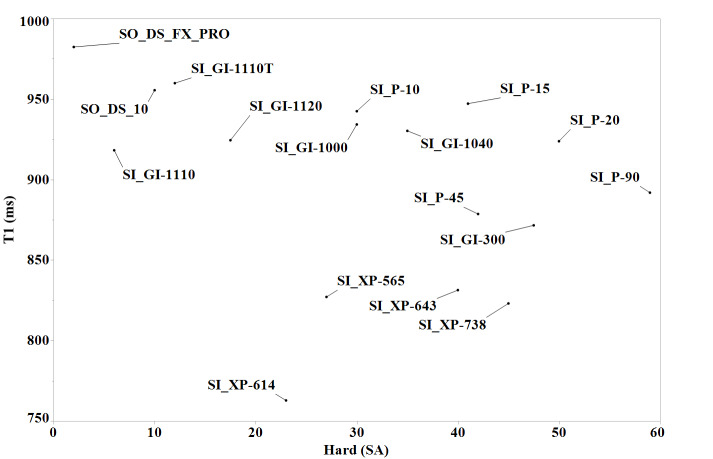
T1 vs Hardness.

**Fig. S11:**
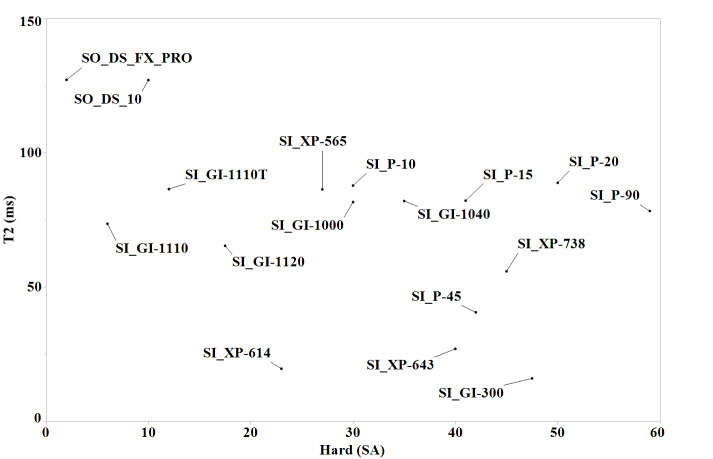
T_2_ vs Hardness.

**Fig. S12:**
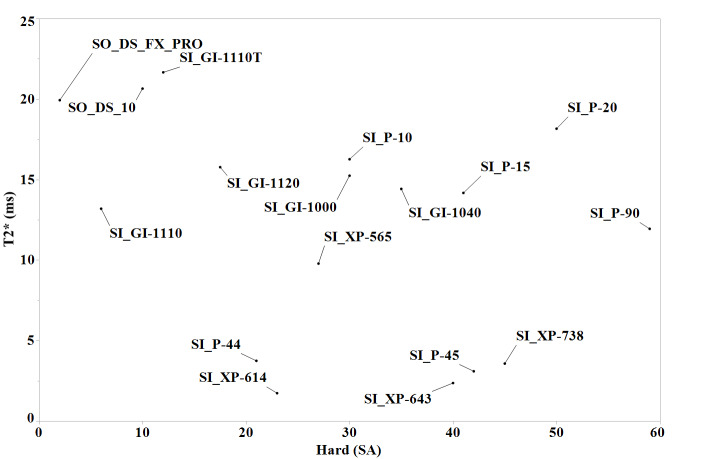
T_2_* vs Hardness.

## 6 Appendix A

**Table A1:** MRI sequences.

T	Sequence	Feld of View (mm)	Resolution *x* × *z* (mm)	TR^a^ (ms)	TE, TI (ms)	Flip Angle (degree)	Average
T_1_	Spin echo inversion recovery	120	128 × 128 0.93	10,000	TE 10.86 TI 10, 18, 32.5, 58.5, 105, 190, 342, 616, 1110, 2000	180 90	1
T_2_	Spin echo	120	256 × 256 0.47	10,000	TE 15, 30, 60, 120, 240, 480, 960	90	1
T_2_*	Gradient echo	120	128 × 128 0.93	1000	TE 2.9, 5.8, 11.6, 23.2,46.4,92.8,185.6	90	8

a (TR) repetition time, (TE) echo time, (TI) inversion time.

**Table A2:** Tissue values reported in literature.

Material^a^	T_1_ (ms)	T_1_ Error (ms)	T_2_ (ms)	T_2_ Error (ms)	T_2_* (ms)	T_2_* Error (ms)	Ref.
Blood	1932	85	275	50			42
Bone cortical	223	11					27
Bone cortical					0.39	0.019	27
Bone cortical (P1.8%) [LC]					2.47	0.323	29
Bone cortical (P1.8%) [SC]					0.318	0.024	29
Bone cortical (P6.8%) [LC]					1.904	0.112	29
Bone cortical (P6.8%) [SC]					0.237	0.037	29
Bone marrow			160				37
Bone marrow	586	73	49	10			32
Bone marrow	371	7.9	133	4.43			30
Bone marrow			40				37
Cartilage	1240	107	36.9	3.81			30
Cartilage			28				37
Cartilage			45				37
Cartilage 0°	1168	18	27	3			42
Cartilage 55°	1156	10	43	2			42
Fat	421	104					36
Fat	385	34	121	20			33
Fat	253	42	68	4			38
Fat			154	9			31
Fat			41				37
Fat breast	450	26	54	9			28
Fat subcutaneous	365	9	133	6.14			30
Fibroglandular	1680	180	71	6			31
Fibroglandular	1445	93	54	9			28
Gray matter	1193	65	109	11			39
Gray matter	1165	113	83	4			35
Gray matter	1820	114	99	7			42
Heart	1471	31	47	11			42
Kidney	1194	27	56	4			42
Kidney cortex	1314	77	47	10			38
Kidney cortex	1142	154	76	7			32
Kidney medulla	1702	205	60	21			38
Kidney medulla	1545	142	81	8			32
Liver	745	65	31	6			38
Liver	809	71	34	4			32
Liver	812	64	42	3			42
Muscle	1509	150					36
Muscle	1295	83	40	3			33
Muscle	1100	59	44	9			38
Muscle	898	33	29	4			32
Muscle	1420	38	31.7	1.9			30
Muscle			27				37
Muscle			44				37
Muscle skeletal	1412	13	50	4			42
Myocardium	1341	42					25
Myocardium			67	5			40
Myocardium [L]			39	5			41
Optic nerve	1083	39	78	5			42
Pancreas	725	71	43	7			32
Prostate	1700	175	80	34			33
Prostate	1597	42	74	9			32
Prostate	1530	498					43
Spinal cord	993	47	78	2			42
Spleen	1232	92	60	19			38
Spleen	1328	31	61	9			32
Uterus cervix	1616	613	83	7			32
Uterus endo	1453	123	59	1			32
Uterus myom	1514	156	79	10			32
White matter	781	61	65	6			39
White matter	728	433	75	3			35
White matter	954	39					26
White matter	1084	45	69	2			42
White matter (V1)					0.216	0.03	34
White matter (V2)					0.358	0.036	34

a SC/LC = short/long compartment of T_2_*; Px.x% = porosity, V*x* = volunteer *x*.

**Table A3:** Sample properties and measured T_1_, T_2_, and T_2_* values.

Print/ Cast^a^	Fabrication^b^	Sample Name^c^	Density (kg/m^3^)	Elong^d^ (%)	TS^d^ (MPa)	Hard^d^ (SA)	T_1_ (ms)	T_1_ Error (ms)	T_2_ (ms)	T_2_ Error (ms)	T_2_* (ms)	T_2_* Error (ms)
Cast	Silicone (add)	SI_XP-614	1240	175.0	4.1	23.0	762.8	36.2	19.5	5.2	1.7	0.5
Cast	Silicone (add)	SI_XP-738	1990	600.0	4.8	45.0	823.0	12.2	55.8	10.5	3.6	0.6
Cast	Silicone (add)	SI_XP-565	1020	ND	ND	27.0	827.1	34.2	86.3	7.0	9.8	1.1
Cast	Silicone (add)	SI_XP-643	1130	700.0	5.0	40.0	831.3	27.6	26.9	8.0	2.4	0.2
Cast	Silicone (cond)	SI_GI-300	1350	160.0	4.1	47.5	871.6	20.6	15.9	7.1	NF	NF
Cast	Silicone (add)	SI_P-45	1120	275.0	5.5	42.0	878.6	7.5	40.5	4.5	3.1	0.3
Cast	Silicone (add)	SI_P-90	1130	415.0	4.1	59.0	891.9	3.5	78.3	6.2	11.9	2.8
Cast	Silicone (cond)	SI_GI-1110	1080	450.0	1.9	6.0	918.2	14.2	73.5	20.9	13.2	0.6
Cast	Silicone (add)	SI_P-20	1080	425.0	3.6	50.0	923.8	5.8	88.8	3.3	18.2	3.4
Cast	Silicone (cond)	SI_GI-1120	1080	475.0	2.7	17.5	924.4	7.0	65.3	2.0	15.8	1.4
Cast	Silicone (cond)	SI_GI-1040	1100	225.0	3.6	35.0	930.3	14.7	82.0	40.2	14.4	2.8
Cast	Silicone (cond)	SI_GI-1000	1090	300.0	3.6	30.0	934.2	12.6	81.6	26.0	15.2	1.9
Cast	Silicone (add)	SI_P-10	1080	450.0	2.4	30.0	942.5	12.7	87.8	24.7	16.3	1.6
Cast	Silicone (add)	SI_P-15	1080	460.0	3.3	41.0	947.2	17.6	82.1	22.1	14.2	1.7
Cast	Silicone (add)	SO_DS_10	1070	1000.0	3.275	10.0	955.5	8.7	127.1	12.3	20.7	4.2
Cast	Silicone (cond)	SI_GI-1110T	1080	450.0	1.89	12.0	959.9	6.0	86.4	3.0	21.7	1.1
Cast	Silicone (add)	SO_DS_FX_PRO	1062	763.0	1.984	2.0	982.4	5.5	127.2	9.7	19.9	2.8
Cast	Silicone (add)	SO_EF-00-20	1070	845.0	1.102	20 (00)	986.2	4.9	136.9	11.3	21.9	2.1
Cast	Silicone (add)	SO_EF-00-35	1070	900.0	1.378	35 (00)	998.3	10.6	148.2	5.5	5.8	0.2
Cast	Silicone (add)	SI_P-44	1090	250.0	4.1	21.0	NS	NS	NS	NS	3.74	0.7
Print	FDM	ST_ABS-M30	1040	7.0	32	NS	NS	NS	NS	NS	19.3	4.5

(Cast) cast, (Print) printed.

b (add) addition cure, (cond) condensation cure, (FDM) fused deposition modeling. In silicone chemistry, the condensation cure uses a tin (Sn) salt that expels water as a reaction by-product, and the addition cure uses a platinum (Pt) reaction that creates an ethyl bridge between the polymer chains.

c Sample names are contractions of the manufacturer and the product name from the data sheet: (3D) 3D Systems, (AL) ALM, (CN) Carbon, (CR) Carbon Resin, (DS) DSM Somos, (ES) EOS, (FL) Formlabs, (FN) ProtoLabs FineLine, (NT) NinjaTek, (HN) Huntsman, (SI) Silicones, Inc., (SO) Smooth-On, Inc., (ST) Stratasys.

d (Elong) elongation, (TS) tensile strength, (Hard) hardness, (SA) Shore A scale, (00) Shore 00 scale. The two SO_EF-00-XX samples were soft “skin-like” materials that were measured on the Shore 00 scale, which covers materials below the range of the Shore A hardness scale.

e (ND) no data, (NS) no signal, (NF) no fit.

**Table A4:** Material properties vs. T_1_, T_2_, andT_2_*.

Property	T_1_	T_2_	T_2_*
Density	No fit	No fit	No fit
Elongation	No fit	*Y* = 22.57 + 0.1122*X* *R*^2^ = 0.523 *p* = 0.0007	No fit
Tensile strength	*Y* = 1036 − 37.27*X* *R*^2^ = 0.593 *p* = 0.002	*Y* = 153.1 − 22.79*X* *R*^2^ = 0.566 *p* = 0.0003	*Y* = 24.36 − 3.673*X* *R*^2^ = 0.430 *p* = 0.003
Hardness	No fit	No fit	No fit

## 7 Appendix S

### Supplementary Information

Materials with no measurable signal using the described methods sorted by type, fabrication method and sample name.

## Appendix

**Table S1:**

**Print/ Cast**	**Fabrication^a^**	**Sample Name^b^**	**Res** **(mm)^c^**	**Density (kg/m3)**	**Elong** **(%)**	**TS** **(Mpa)**	**Hard** **(SA)**	**NS/ NF**
Cast	Polyurethane	HN_RP-6400	ND	1040	251.0	7.88	52	NS
Cast	Polyurethane	SO_FF-ITI6	ND	900	ND	ND	ND	NS
Cast	Polyurethane	SO_SMSH	ND	1036	0.0	ND	ND	NF
Cast	Silicone (add)	SI_P-17	ND	1030	150.0	3.4	16.5	NF
Cast	Silicone (add)	SI_P-268	ND	1300	175.0	5.9	ND	NS
Cast	Silicone (add)	SI_P-50	ND	1300	200.0	5.2	44.5	NS
Cast	Silicone (add)	SI_P-60	ND	1240	200.0	6.2	53.0	NS
Cast	Silicone (add)	SO_SF15	ND	240	ND	ND	ND	NS
Cast	Silicone (cond)	SI_GI-311	ND	1140	150.0	2.2	43.0	NS
Cast	Silicone (cond)	SI_GI-360	ND	1490	100.0	4.5	62.0	NS
Print	3DP	3D_CB_ZB	0.150	1040	20.0	ND	ND	NS
Print	FDM	ST_ABS	0.254	1080	6.0	33	ND	NS
Print	FDM	ST_ABS+P430_R	0.254	1040	6.0	33	ND	NS
Print	FDM	ST_ABS+P430_U	0.178	ND	ND	ND	ND	NS
Print	FDM	ST_ABS-ESD7	0.254	1040	3.0	36	ND	NS
Print	FDM	ST_ABSi	0.254	1080	4.4	37	ND	NS
Print	FDM	ST_ABS-M30i	0.254	1040	4.0	36	ND	NS
Print	FDM	ST_Ny_12_PA	0.254	1160	30.0	46	ND	NS
Print	FDM	ST_PC	0.254	1200	4.8	57	ND	NS
Print	FDM	ST_PC-ABS	0.254	1098	5.0	34	ND	NS
Print	FDM	ST_PC-ISO	0.254	1200	4.0	57	ND	NS
Print	FDM	ST_UM 9085	0.254	1340	5.8	69	NS	NS
Print	PolyJet	ST_FLX2040	0.016	1120	110.0	1.3	37.5	NS
Print	PolyJet	ST_FLX2050	0.016	1120	95.0	1.9	47.5	NS
Print	PolyJet	ST_FLX2060	0.016	1120	75.0	2.5	60.0	NS
Print	PolyJet	ST_FLX2070	0.016	1120	65.0	3.5	70.0	NS
Print	PolyJet	ST_FLX2085	0.016	1120	55.0	5.5	82.5	NS
Print	PolyJet	ST_FLX2095	0.016	1120	40.0	9.8	95.0	NS
Print	PolyJet	ST_FLX930_P	0.038	1120	170.0	0.79	27.0	NS
Print	PolyJet	ST_FLX930_U	0.016	1120	170.0	0.8	27.0	NS
Print	PolyJet	ST_FLX9540	0.016	1120	100.0	1.3	40.0	NS
Print	PolyJet	ST_FLX9550	0.016	1120	80.0	2	53.5	NS
Print	PolyJet	ST_FLX9560	0.016	1120	60.0	2.8	63.5	NS
Print	PolyJet	ST_FLX9570	0.016	1120	50.0	3.8	74.0	NS
Print	PolyJet	ST_FLX9585	0.016	1120	35.0	6	86.0	NS
Print	PolyJet	ST_FLX9595	0.016	1120	27.0	9	95.5	NS
Print	PolyJet	ST_RGD5131DM	0.038	1170	25.0	55	ND	NS
Print	PolyJet	ST_RGD5150	0.016	ND	18.0	45	ND	NS
Print	PolyJet	ST_RGD525	0.016	1170	10.0	70	ND	NS
Print	PolyJet	ST_RGD810	0.038	1180	10.0	50	ND	NS
Print	PolyJet	ST_RGD835_PG	0.016	1170	10.0	50	NS	NS
Print	PolyJet	ST_RGD835_SC	0.038	ND	ND	ND	NS	NS
Print	PolyJet	ST_RGD8455	0.016	ND	20.0	35	NS	NS
Print	PolyJet	ST_RGD8460	0.016	ND	35.0	25	NS	NS
Print	Polyjet	ST_RGD875	0.016	1170	10.0	50	NS	NS
Print	SLA	3D_Acc_25	0.102	1190	13.0	38	ND	NS
Print	SLA	3D_Acc_5530	0.051	1250	1.3	47	ND	NS
Print	SLA	3D_Acc_60	0.051	1210	5.0	58	ND	NS
Print	SLA	3D_Acc_XW200	0.102	1180	7.0	46	ND	NS
Print	SLA	CN_CE 220	0.150	1100	3.0	90	ND	NS
Print	SLA	CN_PR 25	0.150	1100	3.0	42	ND	NS
Print	SLA	CN_RPU 70	0.150	1010	90.0	42	ND	NS
Print	SLA	CR_EPU40	0.150	1000	190.0	6	68	NS
Print	SLA	DS_9120_PG	0.051	1130	15.0	30	ND	NS
Print	SLA	DS_9120_PL	0.051	1130	15.0	30	ND	NS
Print	SLA	DS_NanoT	0.051	1650	0.7	66.3	ND	NS
Print	SLA	DS_NeXt	0.102	1170	8.0	41	ND	NS
Print	SLA	DS_PG_18420	0.102	1160	12.0	42.7	ND	NS
Print	SLA	DS_Ws_XC_11122	0.102	1120	11.0	47	ND	NS
Print	SLA	FL_BLK	0.025	1090	6.0	64.6	ND	NS
Print	SLA	FL_CAST	0.025	1090	13.0	11.6	ND	NS
Print	SLA	FL_CLR	0.025	1090	6.0	64.6	ND	NS
Print	SLA	FL_DENT	0.050	1090	ND	ND	ND	NS
Print	SLA	FL_DENTM	0.025	ND	5.0	61	ND	NS
Print	SLA	FL_DUR	0.050	ND	67.0	31.8	ND	NS
Print	SLA	FL_FLEX	0.050	1090	85.0	8.5	85	NS
Print	SLA	FL_GREY	0.025	1090	6.0	64.6	ND	NS
Print	SLA	FL_HITMP	0.025	1100	2.0	51	ND	NS
Print	SLA	FL_TOUGH	0.050	1090	24.0	41.3	ND	NS
Print	SLA	FL_WHT	0.050	1090	6.0	64.6	ND	NS
Print	SLA	FN_MF_G	0.025	1170	6.1	44.9	ND	NS
Print	SLA	HN_RS-7820	0.051	1130	8.0	35.8	ND	NS
Print	SLS	3D_Duraf	0.102	1200	4.5	31	ND	NS
Print	SLS	AL_PA_614GS	0.102	1220	9.0	51	ND	NS
Print	SLS	AL_PA_650	0.102	1020	24.0	ND	ND	NS
Print	SLS	AL_PA_850	0.102	1030	51.0	48	ND	NS
Print	SLS	ES_PA_1102B	0.150	990	45.0	48	ND	NS
Print	SLS	ES_PA_2200	0.060	900	20.0	45	ND	NS
Print	SLS	ST_VJ_PXL	0.102	1000	0.2	14.2	NS	NS

a (add) Addition cure, (SLS) Selective Laser Sintering, (SLA) Stereo Lithography Additive, (FDM) Fused Deposition Modeling, (3DP) 3D Printing.

b (3D) 3D Systems, (AL) ALM, (CN) Carbon, (CR) Carbon Resin, (DM) DSM Somos, (ES) EOS, (FL) Formlabs, (FN) Fineline Microfine Green, (HN) Huntsman, (SI) Silicones, Inc., (SO) Smooth-On, Inc., (ST) Stratasys.

c (ND) No data, (NS) No-Signal, (NF) No-Fit.

### Statistical Analysis

Note: The No-Data (ND) entries in Table S2 result in no plot labels for SI_P-44 on T_1_ and T_2_ plots, no SI_GI-300 on T_2_* plots, and no SI_XP-565 for Elongation or Tensile Strength plots.
